# Inactivation Effect of Germination Combined with Cold Plasma Treatment on *Bacillus licheniformis* Spores

**DOI:** 10.3390/foods12234319

**Published:** 2023-11-29

**Authors:** Jichao Huang, Kairan Sheng, Yali Zhang, Mengmeng Song, Ahtisham Ali, Tianran Huang, Ming Huang

**Affiliations:** 1 College of Engineering, Nanjing Agricultural University, Nanjing 210095, China; 2 College of Food Science and Technology, Nanjing Agricultural University, Nanjing 210095, China

**Keywords:** *Bacillus licheniformis*, spores, cold plasma, germination, inactivation

## Abstract

Food spoilage, primarily caused by spore-forming bacteria, has become a critical concern since it results in substantial economic losses within the food industry. Past investigations have successfully identified *Bacillus licheniformis* as the main bacterium responsible for spoilage in roast chicken. In this study, we screened a new sterilization combination from 16 germinants and 4 cold plasma conditions, respectively. Among them, the combination of “A”GFNa-1 (composed of 60 mmol/L L-alanine, 10 mmol/L D-glucose, 10 mmol/L D-fructose, and 1 g/L NaCl) with cold plasma treatment (packed with 100% argon at 70 kV) proved effective in deactivating *B. licheniformis* spores, resulting in a reduction of approximately 2.1 log CFU/mL. Furthermore, we exposed the spores to different conditions: CK (no germination, no cold plasma), MF (germination only), CP (no germination, 100% argon packed, 70 kV cold plasma treatment for 3 min), and MF + CP (germination for 5 h, 100% argon packed, 70 kV cold plasma treatment for 3 min). The results of heat inactivation and dipicolinic acid (DPA) release rate demonstrated that cold plasma treatment effectively inactivated both spores and vegetative cells without inducing germination. Additionally, the reduced survival under hyperosmotic conditions and the presence of distinct red fluorescence patterns observed through confocal laser scanning microscopy (CLSM) collectively suggest that cold plasma treatment disrupts the inner membrane structure and leads to the inactivation of *B. licheniformis*. Overall, our findings indicate a spore clearance rate of 99.2% and suggest that the combination of efficient germinants and cold plasma treatment holds promise as a viable approach to mitigate spore contamination in the food industry.

## 1. Introduction

*Bacillus licheniformis* was first isolated from food ingredients by Richmond and Fields in 1966 [1]. Subsequently, it was also discovered in semi-canned meat, chicken carcasses, and hamburgers [2,3]. *B. licheniformis* is a Gram-positive, facultative anaerobic endospore-producing bacterium. It has been widely distributed in natural environments including water and soil [4]. *B. licheniformis* can contaminate food and produce proteases, amylases, and surfactants that can cause foodborne intoxication [5]. Moreover, *B. licheniformis* can survive as dormant spores under adverse environmental conditions, posing challenges for their eradication in the food industry [6]. There is an increasing demand to identify effective, high-quality, and safe thermal sterilization alternatives for the inactivation of bacterial spores [7].

Several studies in the scientific literature have reported the enhanced susceptibility of germinating spores to inactivation [8,9,10,11]. Spore germination can be triggered by exposure to various molecules such as amino acids, sugars, and cell wall muropeptides, which can bind to receptors on the inner spore membrane [12,13]. In a systematic study, Setlow et al. [14] summarized several elements of spore germination on a signal reception including dipicolinic acid (DPA) release, peptidoglycan cortex hydrolysis, and metabolic recovery. Spore germination is an irreversible process involving a series of physiological and biochemical reactions, and germinated spores have reduced resistance [15]. 

Heat sterilization has proven to be ineffective in controlling highly heat-resistant spores in the food processing industry [16]. Previous studies have shown that high-temperature heat treatment can inactivate spores but can also have negative effects on food quality [17]. Therefore, there is a growing interest in finding ways to inactivate spores while also improving the sensory qualities of processed foods. Compared to conventional heat treatment, a more efficient approach is to promptly inactivate spores once germination occurs [18]. Several studies have demonstrated the effectiveness of emerging and alternative methods such as pressure-assisted thermal sterilization [19], high-pressure carbon dioxide [20], supercritical fluid carbon dioxide [21], and cold plasma treatment for food sterilization [22]. Among these methods, cold plasma treatment has received considerable attention due to its simple design, reduced water usage, low operational costs, and promising potential for spore inactivation [23]. 

Cold plasma sterilization technology, as an emerging non-thermal processing method, utilizes ionized gases to generate abundant reactive species, achieving efficient disinfection and sterilization. Cold plasma has emerged as a promising non-thermal technology for food sterilization in the past decade [23,24]. In cold plasma, the presence of reactive species such as ultraviolet (UV) photons, reactive neutral species, and charged particles is believed to be responsible for the effective inactivation of microorganisms [20,25]. Numerous studies have shown that cold plasma can effectively inactivate various spore-forming bacteria [20]. Van Bokhorst-van de Veen et al. [26] indicated that nitrogen-based cold plasma treatment has a biocidal effect on *Bacillus cereus*, *Bacillus atrophaeus*, and *Geobacillus stearothermophilus* spores. Wang et al. [25] observed that cold atmospheric plasma treatments of more than 3 min cause significant visible damage to the structures and germination proteins of *Bacillus subtilis* spores. However, limited research has been conducted on the inhibitory effect of cold plasma specifically on *B. licheniformis*, highlighting the need for further investigation.

This study aimed to evaluate the effects of cold plasma on the germination and inactivation of *B. licheniformis* spores, as evidenced by growth curves and residual spore counts, respectively. The mechanisms underlying the inactivation of *B. licheniformis* spores by cold plasma were investigated through DPA release, survival rates in a hypertonic medium, and confocal laser scanning microscopy (CLSM). The objective of this research was to examine the inactivation effects and mechanisms of cold plasma on *B. licheniformis* spores in roasted chicken. The findings of this study will provide a theoretical foundation for controlling *B. licheniformis* spores and preventing spore contamination in poultry meat. 

## 2. Materials and Methods

### 2.1. Bacterial Strains Used in This Study

The *B. licheniformis* strain used in this study was isolated from commercially available roasted chicken purchased from Nanjing Huangjiaoshou Food Science and Technology Co., Ltd. (Nanjing, China). The isolation procedure followed the method described by Li et al. [27] with minor modifications. The strain was identified through 16S rRNA sequence analysis, and the sequence data were deposited in the National Center for Biotechnology Information (NCBI) database with the accession number MT807915. The strain was inoculated into Luria Bertani (LB) broth and incubated overnight in a constant-temperature oscillating incubator (Iversen Biotechnology Co., Ltd, Hebei, China) at 37 °C. This bacterial suspension was transferred to LB containing 25% glycerol and stored at −80 °C until used for further analysis.

### 2.2. Preparation and Purification of Spore Suspension

Spore suspensions of *B. licheniformis* were prepared and purified as previously described by Li et al. [27]. The activated culture of *B. licheniformis* was inoculated onto the manganese (Mn^2+^)-amended nutrient agar (50 mgMn^2+^, pH 7.0–7.2), which was then incubated at 37 °C for 5~7 days. Spores from cultures were collected using a refrigerated high-speed centrifuge at 7000 rpm, 15 min, 4 °C (Allegra-64R, Beckman Coulter, Inc., Brea, CA, USA), and washed three times with cold sterile distilled water. After being preserved at 4 °C for 24 h, the spore suspensions were heat-shocked at 80 °C for 20 min to inactivate vegetative cells. Bright green spores were observed to be over 95% pure under the fluorescent microscopy (Scope.A1, Carl Zeiss AG, Oberkochen, Germany). The spore suspension was then adjusted to the desired concentration with sterile distilled water, stored at 4 °C, and used within one month. 

### 2.3. Preparation of B. licheniformis Germinants

Nutritional agar (NA), LB broth, D-glucose (G), D-fructose (F), D-galactose (Gal), L-alanine (Ala), L-asparagine (Asp), L-valine (Val), L-proline (Pro), L-lysine (Lys), and L-histidine (His) were obtained from Dingbei Biotechnology Co., Ltd. (Nanjing, China). NaCl, KCl, MgCl_2_, CaCl_2_, and CH_3_COONa were obtained from Sinopharm Chemical Reagent Co., Ltd. (Shanghai, China). DPA was obtained from Sigma-Aldrich Trading Co., Ltd. (Shanghai, China). All chemicals used in this study were analytical grade. Different germinants were weighed and dissolved in Tris-HCl buffer (25 mmol/L, pH 7.4) to prepare the concentrations shown in Table 1.

### 2.4. Selection of B. licheniformis Germinants

#### 2.4.1. Measurement of OD600

The optical density at 600 nm (OD_600_) gradually dropped by up to 60% during germination [18]. The decrease in OD_600_ can be used for evaluating spore germination. Spore suspensions were heat-activated at 75 °C for 15 min and centrifuged (7000 rpm, 15 min, 4 °C) to collect the precipitates. Germinants, as explained in Section 2.3, were prepared at 37 °C and added to the spores. The mixture was then vigorously vortexed until complete homogenization was achieved. The OD_600_ of these mixtures was measured every 5 min using an automated growth curve detector (FP-1100-C, Oy Growth Curves Ab Ltd., Helsinki, Finland).

#### 2.4.2. Thermal Inactivation and Germination Rate of Spores

The germination process leads to a reduction in the heat resistance of spores, as reported by Liao et al. [23]. Therefore, the effect of germinants on spore germination could be measured as thermal inactivation of spores. Spore suspensions, before and after treatment with germinants as described in Section 2.4.1, were exposed to a water bath at 80 °C for 20 min and immediately cooled. Afterward, 100 μL of suitable diluents was inoculated onto sterile NA and incubated at 37 °C for 24 h in a microbiological incubator (B1-150A, STIK, Shanghai, China). The surviving colonies were then enumerated, and the germination rate of spores was calculated as follows:

(1) $$\;\mathrm{Germination}\;\mathrm{rate}\;\mathrm{of}\;\mathrm{spores}=-\log\left(\frac{N_{t}}{N_{0}}\right)$$

In Equation (1), *N_t_* is the number of surviving colonies after germinants and heat treatment, and *N*_0_ is the initial number of colonies after germinants and before heat treatment.

#### 2.4.3. Determination of DPA Release Rate

It has been established that spores release almost all of their DPA from the spore core upon germination, as reported by Zhu et al. [8]. When released, DPA forms complexes with terbium (Tb^3+^) and fluoresces. Hence, monitoring fluorescence intensities can be used to assess spore germination. Different concentrations of DPA were mixed with CH_3_COONa buffer (pH 5.6) containing TbCl_3_-6H_2_O (1 mol/L) obtained from Titan Scientific Co., Ltd., Shanghai, China. A standard curve was generated by measuring the fluorescence of the DPA-TbCl_3_-6H_2_O complex at the optimum excitation wavelength (272 nm) and emission wavelength (542 nm). Spore suspensions, both before and after treatment with germinants as described in Section 2.4.1, were centrifuged at 13,200 rpm and 4 °C for 5 min, and the collected supernatants were filtered using a 0.45 μm filter membrane. The filtered supernatants were mixed with CH_3_COONa buffer (1 mol/L TbCl_3_-6H_2_O, pH 5.6) in a 96-well plate (1:1 dilution), and the fluorescence was measured using a multifunctional microplate reader (M2e, Molecular Devices, San Jose, CA, USA). The percentage of DPA release (% of initial) was calculated using the following equation:

(2) $$\mathrm{DPA}\;\mathrm{release}\;\left(\%\;\mathrm{of}\;\mathrm{initial}\right)=\frac{D_{t}}{D_{0}}\times 100\%$$

In Equation (2), *D_t_* is the amount of DPA released by the treatment group, and *D*_0_ is the amount of initial total DPA.

### 2.5. Selection of Cold Plasma Conditions

To screen the optimal inactivation conditions, the best germinant “A”GFNa-1 (60 mmol/L L-alanine, 10 mmol/L D-glucose, 10 mmol/L D-fructose, 1 g/L NaCl) was employed in combination with cold plasma treatment. Cold plasma treatment was carried out following the procedure outlined by Wang et al. [25], with a maximum voltage limit of 70 kV to ensure the quality of the chicken meat was not compromised. Spore suspensions, treated with “A”GFNa-1 as described in Section 2.4.1, were adjusted to a concentration of 1 × 10^4^ CFU/mL, vortexed homogeneously, and incubated at 37 °C for 5 h. Germinated spore suspensions (50 μL) were dispensed onto sterile coverslips (20 mm × 20 mm) and placed in Petri dishes, which were then transferred to sealed boxes. The experimental groups included five categories: air (100% air, no cold plasma), Ar (100% argon, no cold plasma), C-50 (100% argon packed, 50 kV cold plasma), C-60 (100% argon packed, 60 kV cold plasma), and C-70 (100% argon packed, 70 kV cold plasma). Subsequently, the samples were exposed to high-voltage electric field cold plasma (CPS-I, Yirun Plasma Technology Co., Nanjing, China) for 3 min and stored at 37 °C for 24 h. Surviving spores were collected from coverslips, adjusted to a specific concentration using sterile saline, and then the thermal inactivation of spores was determined as described in Section 2.4.2.

### 2.6. Inactivation Effect and Mechanism of Cold Plasma on B. licheniformis

#### 2.6.1. Thermal Inactivation of *B. licheniformis*

The spore suspensions were divided into four groups: CK (no germination, no cold plasma), MF (germination only), CP (no germination, 100% argon packed, 70 kV cold plasma for 3 min), and MF + CP (germination for 5 h, 100% argon packed, 70 kV cold plasma for 3 min), as outlined in Section 2.5. The thermal inactivation was then measured following the protocol described in Section 2.4.2.

#### 2.6.2. *B. licheniformis* Growth Curve Determination

The spore suspensions, both before and after treatments, were inoculated into LB and homogenized by vortexing. OD_600_ of these mixtures was subsequently measured every 30 min using an automated growth curve detector (FP-1100-C, Oy Growth Curves Ab Ltd., Helsinki, Finland) at 37 °C for 24 h.

#### 2.6.3. DPA Release Rate of *B. licheniformis* Spores

The spore suspensions, both before and after treatments mentioned in Section 2.4.3, were centrifuged and filtered. Subsequently, the filtered solutions were subjected to measurement using a multifunctional microplate reader.

#### 2.6.4. Survival Status of Spores of *B. licheniformis* in Hypertonic Medium

The spore suspensions, both before and after treatments, were inoculated into a sterile hypertonic medium (composed of 1 mol/L NaCl and 50 mmol/L L-glucose). The inoculated suspensions were subsequently incubated at 37 °C for 24 h and the survival rate was observed and calculated.

#### 2.6.5. Confocal Laser Scanning Microscope (CLSM) Analysis

Spore suspensions, both before and after treatments, were adjusted to a concentration of 10^6^ CFU/mL and fixed on sterile slides. The slides were then stained with membrane-impermeable staining pyridinium iodide dyes (PI, 3 μL in 1 mL, Invitrogen Trading Co., Ltd., Shanghai, China) and stored in the dark at room temperature for 45 min [18]. The fluorescence emitted by the stained samples was observed using a CLSM with a 63 × oil immersion objective (TCS SP8, Leica Microsystems, Wetzlar, China), utilizing an excitation wavelength of 490 nm and an emission wavelength of 635 nm.

### 2.7. Statistical Analysis

Experiments were carried out in triplicate and averages were recorded. Data were imported into the Statistical Analysis System (SAS, Release 8.1; SAS Institute Inc., Cary, NC, USA) for statistical analysis. One-way ANOVA was used to assess differences in germination rate and DPA release rate between treatment combinations. Two-way ANOVA was used to assess the effect of factors such as thermal inactivation and hypertonic medium on colony numbers. Duncan’s multiple range tests identified significant differences between treatments (*p* < 0.05) following these analyses. Origin software (version 9.0; OriginLab Inc., Northampton, MA, USA) was used for image processing. 

## 3. Results and Discussion

### 3.1. Selection of Germinants

**Figure 1:**
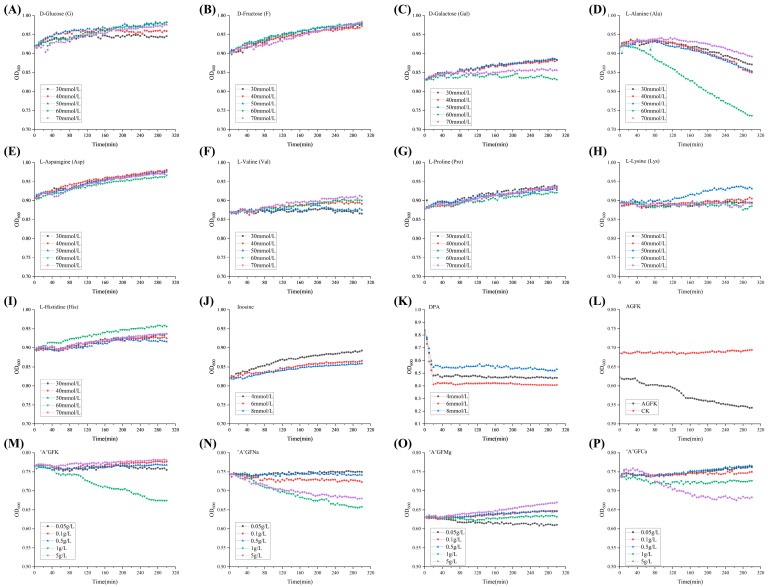
Preliminary screening of the germination effect of germinants. (**A**–**I**) Growth curves of spores under the influence of different concentrations (30 mmol/L, 40 mmol/L, 50 mmol/L, 60 mmol/L, 70 mmol/L) of D-glucose (G), D-fructose (F), D-galactose (Gal), L-Alanine (Ala), L-Asparagine (Asp), L-Valine (Val), L-Proline (Pro), L-Lysine (Lys), and L-Histidine (His). (**J**,**K**) Growth curves of spores under the influence of inosine at concentrations of 4 mmol/L, 6 mmol/L, and 8 mmol/L, and DPA at concentrations of 4 mmol/L, 6 mmol/L, and 8 mmol/L. (**L**) Growth curve of spores under the influence of AGFK (10 mmol/L Asp, 10 mmol/L G, 10 mmol/L F, 50 mmol/L KCl). (**M**–**P**) Growth curves of spores under the influence of Ala (60 mmol/L), G (10 mmol/L), and F (10 mmol/L) in the presence of KCl/NaCl/MgCl_2_/CaCl_2_ at concentrations of 0.05 g/L, 0.1 g/L, 0.5 g/L, 1 g/L, and 5 g/L. For detailed information on the treatment groups, please refer to Table 1.

As illustrated in Figure 1A–C, no significant facilitatory effect (*p* > 0.05) of the three D-type sugars (G, F, Gal) on *B. licheniformis* spore germination was observed. Among the different concentrations of L-type amino acids (Ala, Asp, Val, Pro, Lys, His) and inosines (Figure 1D–J), spores exposed to 60 mmol/L Ala showed significant (*p* < 0.05) promotion of germination over inosine concentration. Additionally, three concentrations of DPA (4 mmol/L, 6 mmol/L, and 8 mmol/L) significantly promoted (*p* < 0.05) the induced germination of *B. licheniformis* spores (Figure 1K). These findings suggest that DPA can positively influence spore germination, particularly in cases where nutrient germinants are less effective. According to Francis and Sorg [28], this DPA directly activated the cortex lytic enzyme (CWIJ), which induced the germination of spores. However, the safety of DPA remains a challenge when applied to food matrices. Notably, spores germinated rapidly when exposed to the germinant combination AGFK (10 mmol/L Asp, 10 mmol/L G, 10 mmol/L F, 50 mmol/L KCl) (Figure 1L), while individual components A, G, and F exhibited no significant effect (*p* > 0.05). To further investigate and optimize the germinant combinations, 60 mmol/L Ala was combined with varying concentrations of K^+^, Na^+^, Mg^2+^, and Ca^2+^ (Figure 1M–P). Treatments with 1 g/L K^+^, 1 g/L and 5 g/L Na^+^, 0.05 g/L Mg^2+^, and 5 g/L Ca^2+^ resulted in a significantly (*p* < 0.05) increased spore germination, while certain additions of cations displayed inhibitory effects. These observations may be attributed to the expression of ion channels during germination [29]. Consequently, the following eight germinants were selected for subsequent experiments: Ala (60 mmol/L), AGFK (10 mmol/L Asp, 10 mmol/L G, 10 mmol/L F, 50 mmol/L KCl), “A”GFNa-1 (60 mmol/L Ala, 10 mmol/L G, 10 mmol/L F, 1 g/L NaCl), “A”GFNa-5 (60 mmol/L Ala, 10 mmol/L G, 10 mmol/L F, 5 g/L NaCl), “A”GFMg-0.05 (60 mmol/L Ala, 10 mmol/L G, 10 mmol/L F, 0.05 g/L MgCl_2_), “A”GFCa-1 (60 mmol/L Ala, 10 mmol/L G, 10 mmol/L F, 1 g/L CaCl_2_), and “A”GFCa-5 (60 mmol/L Ala, 10 mmol/L G, 10 mmol/L F, 5 g/L CaCl_2_).

**Figure 2:**
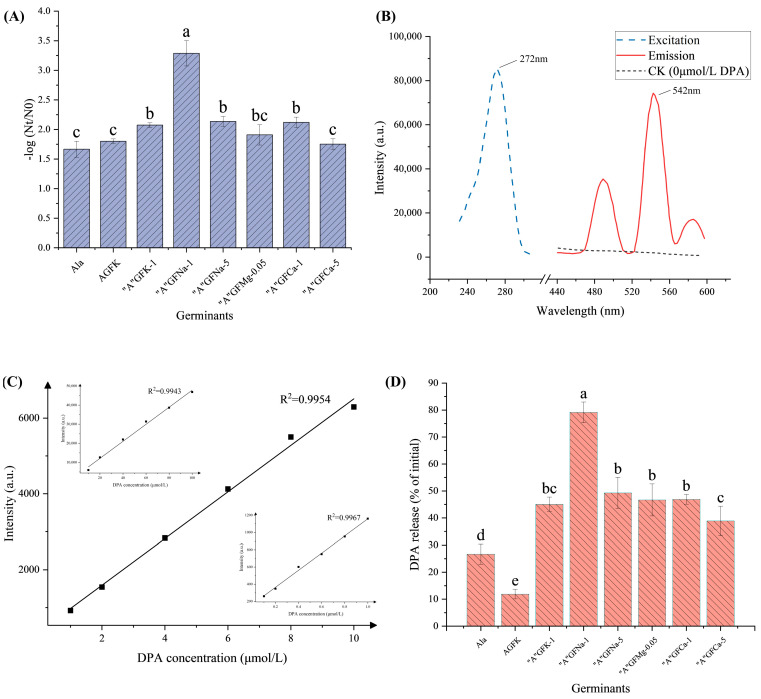
Further screening of the germination effect of germinants. (**A**) Thermal inactivation and germination rate of spores under the influence of eight different germinants: Ala (60 mmol/L), AGFK (10 mmol/L Asp, 10 mmol/L G, 10 mmol/L F, 50 mmol/L KCl), “A”GFNa-1 (60 mmol/L Ala, 10 mmol/L G, 10 mmol/L F, 1 g/L NaCl), “A”GFNa-5 (60 mmol/L Ala, 10 mmol/L G, 10 mmol/L F, 5 g/L NaCl), “A”GFMg-0.05 (60 mmol/L Ala, 10 mmol/L G, 10 mmol/L F, 0.05 g/L MgCl_2_), “A”GFCa-1 (60 mmol/L Ala, 10 mmol/L G, 10 mmol/L F, 1 g/L CaCl_2_), and “A”GFCa-5 (60 mmol/L Ala, 10 mmol/L G, 10 mmol/L F, 5 g/L CaCl_2_). (**B**) Fluorescence excitation and emission spectra of the TbCl_3_·6H_2_O-DPA complex. (**C**) Standard curve depicting the relationship between DPA concentration and fluorescence intensity. (**D**) Effects of the eight different germination factors on DPA release for *B. licheniformis* spores. Detailed information regarding the treatment groups can be found in Table 1. Error bars represent the standard deviations of the mean (*n* = 3). Means with different letters were significantly different (*p <* 0.05).

The results obtained from the thermal inactivation analysis following heat treatment of the spores showed a positive correlation with the germination rate. As illustrated in Figure 2A, the thermal inactivation of spores treated with “A”GFK-1, “A”GFNa-5, and “A”GFCa-1 (mean = 2.1 log CFU/mL, *p* > 0.05) was significantly (*p* < 0.05) higher than that of groups containing Ala, AGFK, and “A”GFCa-5 (mean = 1.74 log CFU/mL, *p* > 0.05). Notably, “A”GFNa-1 caused the most germination at approx. 3.2 log CFU/mL.

Furthermore, the DPA release rate results are consistent with the observed thermal inactivation results. As illustrated in Figure 2B, the TbCl_3_-6H_2_O complex with DPA displayed peak excitation and emission wavelengths at 272 nm and 542 nm, respectively. Standard curves displaying the relationship between DPA concentration (concentration 0.1–1 μmol/L, 1–10 μmol/L, 10–100 μmol/L) and fluorescence intensity were plotted (R^2^ = 0.9965, 0.9954, 0.9941) (Figure 2C). Among the tested combinations, “A”GFNa-1 consistently demonstrated the highest performance, with a DPA release rate of up to 80%. The DPA release rates of the spores ranged between 40% and 50% following treatment with “A”GFK-1, “A”GFNa-5, “A”GFMg-0.05, “A”GFCa-1, and “A”GFCa-5 (Figure 2D). However, in comparison to the 72% DPA release rate reported by Zhu et al. [30], our study only achieved a 10% release of DPA with the AGFK treatment. This disparity may be attributed to differences in spore strains and inner membrane receptors. In summary, the “A”GFNa-1 germinant was chosen for further experimentation.

### 3.2. Selection of Cold Plasma Conditions

**Figure 3:**
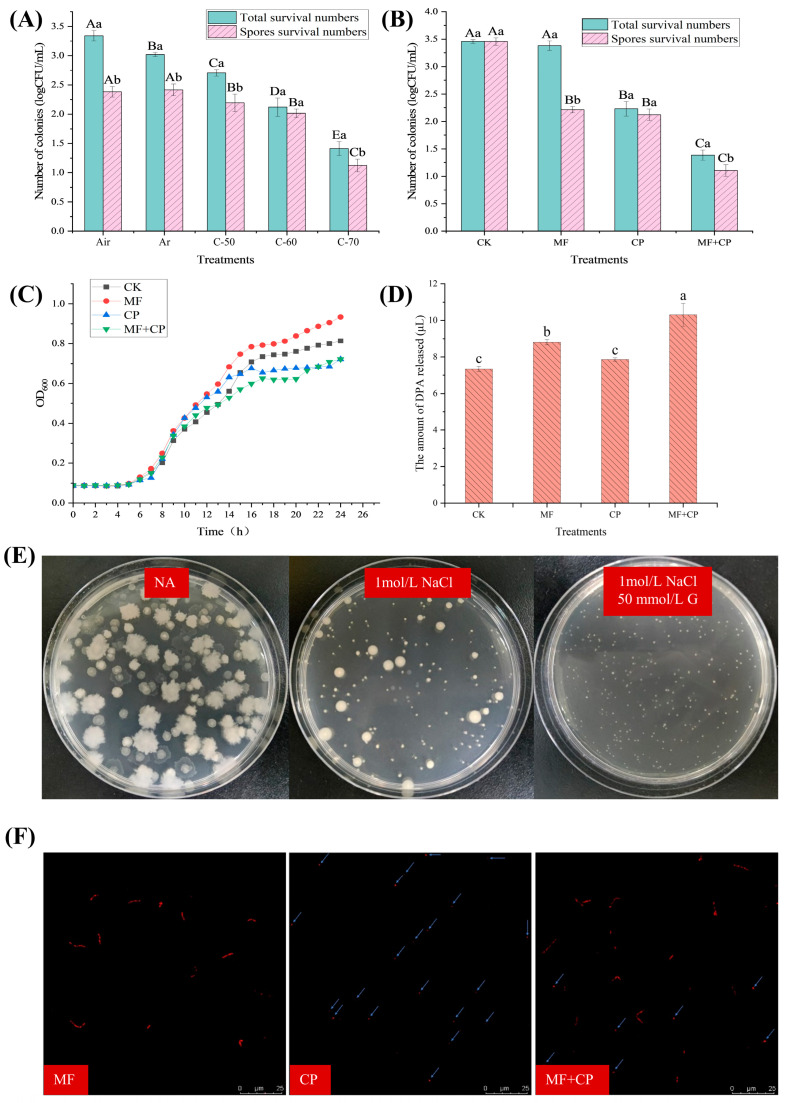
(**A**) Thermal inactivation of *B. licheniformis* under different conditions: air (100% air, no cold plasma), Ar (100% argon, no cold plasma), C-50 (100% argon packed, 50 kV cold plasma), C-60 (100% argon packed, 60 kV cold plasma), and C-70 (100% argon packed, 70 kV cold plasma). (**B**) Thermal inactivation of *B. licheniformis* under different treatments: CK (no germination, no cold plasma), MF (germination only), CP (no germination, 100% argon packed, 70 kV cold plasma for 3 min), and MF + CP (germination for 5 h, 100% argon packed, 70 kV cold plasma for 3 min). (**C**) Determination of *B. licheniformis* growth curves under different treatments: CK, MF, CP, and CP + MF. (**D**) DPA release rate of *B. licheniformis* spores under different treatments: CK, MF, CP, and CP + MF. (**E**) Survival status of *B. licheniformis* spores in hypertonic medium with different conditions: NA, NA + 1 mol/L NaCl, and NA + 1 mol/L NaCl + 50 mmol/L glucose. (**F**) Analysis of *B. licheniformis* spores using confocal laser scanning microscope (CLSM) under different treatments: MF, CP, and MF + CP. Error bars represent the standard deviations of the mean (*n* = 3). Upper case letters indicate significant differences between groups (*p* < 0.05) and lowercase letters indicate significant differences within groups (*p* < 0.05).

The numbers of surviving *B. licheniformis* under different cold plasma treatment conditions are indicated in Figure 3A. As the cold plasma voltage increased, there was a significant decrease (*p* < 0.05) in the number of surviving *B. licheniformis*. When the voltage reached 70 kV, only 1.41 log CFU/mL remained. It has been reported that after entering the germination stage, spores gradually lose their heat resistance [8]. Therefore, a certain level of heat treatment (80 °C, 20 min) can only kill vegetative cells, allowing for the enumeration of surviving spores [18]. In Figure 3A, the number of surviving *B. licheniformis* vegetative cells was significantly lower (*p* < 0.05) in Ar compared to air; however, there was no significant difference (*p* > 0.05) in spore viability. The results indicate that Ar treatment can effectively inactivate vegetative cells of *B. licheniformis* but has little effect on its spores. However, argon cold plasma significantly decreased (*p* < 0.05) the viability of *B. licheniformis* spores compared to the control group without cold plasma exposure. The spore count in the C-70 group was 1.12 log CFU/mL, which was 1.27 log CFU/mL lower (*p* < 0.05) than that of the control group (2.39 log CFU/mL). The viability of spores was also reduced by C-50 and C-60 treatments to a lesser degree. The experimental results show that argon cold plasma has a killing effect on *B. licheniformis* spores and reached its optimal level at a voltage of 70 kV.

### 3.3. Inactivation Effect of Germinants Combined with Cold Plasma on B. licheniformis

The survival numbers of *B. licheniformis* and its spores after different treatments are described in Figure 3B. The number of surviving spores in the MF group was significantly lower (*p* < 0.05) than that in the CK group, with a difference of approximately 1.4 log CFU/mL. However, the total survival numbers of *B. licheniformis* in the two groups had no significant differences (*p* > 0.05). This indicates that “A”GFNa-1 induced 1.4 log CFU/mL spore germination, and all the uninduced spores could also germinate and grow successively in NA. Additionally, the number of vegetative cells and spores of *B. licheniformis* in the MF + CP group was significantly lower (*p* < 0.05) compared to the MF group. This result further confirms that cold plasma can inactivate both vegetative cells and spores of *B. licheniformis*. Meanwhile, the spore counts in the MF + CP group were significantly (*p* < 0.05) lower than those in the CP group, with a difference of about 1.4 log CFU/mL. This suggests a synergistic effect of the germination agent “A”GFNa-1 and cold plasma on spore inactivation.

### 3.4. Mechanism of Cold Plasma Inactivation of B. licheniformis Spores

The growth curves of spores under cold plasma treatment are shown in Figure 3C. There was an initial period of growth retardation (approx. 5 h) before the spores germinated into vegetative cells and began to proliferate, and no substantial differences were observed between the treatment groups. Typically, spore germination is accompanied by an decrease in OD_600_ values, while the growth and proliferation of vegetative cells are associated with an increase in OD_600_ values [18]. However, in our study, the lack of a significant decrease in OD_600_ values was inconsistent with the previous findings. This discrepancy may be attributed to the low initial spore concentration and the limited sensitivity of the instrument in detecting subtle changes in OD_600_ values. Figure 3C illustrates variations in the growth phase of germinating spores among the treatment groups. The OD_600_ of the MF group exhibited faster growth in the later stage and achieved the highest proliferation within 24 h. This could be attributed to the partial germination of the MF group into vegetative cells, resulting in a faster proliferation rate compared to the CK group. Conversely, the growth of germinating spores in the MF + CP group was delayed compared to the MF and CP groups, respectively. This can be explained by the fact that cold plasma treatment resulted in a lower initial spore count, thus affecting the growth rate and proliferation of *B. licheniformis*. Furthermore, it can be seen that the combination of cold plasma treatment and germinants had a stronger bactericidal effect. The OD_600_ values of the CK group, without cold plasma treatment, were lower than those of the CP and MF + CP groups during the 9–13 h and 9–15 h periods, respectively, and then remained at a higher level. These results suggest that cold plasma treatment may have broken the dormancy of some spores, resulting in earlier germination and metabolism in the CP and MF + CP groups. Collectively, the spore growth curve showed that cold plasma treatment had a significant inactivating effect on both *B. licheniformis* spores and their nutrients. It may have partially disrupted spore dormancy, enabling them to enter germination and metabolism earlier. However, further research is required to elucidate the underlying mechanisms of this effect.

DPA is a specific molecule found in spores that plays a crucial role in maintaining their heat resistance. It functions by binding with calcium ions to form a stable complex, which protects the DNA of spores from heat-induced damage. During spore germination, DPA is released from the spore core, resulting in the loss of heat resistance [31]. Thus, the release of DPA can serve as an indicator of spore germination. As shown in Figure 3D, the amounts of DPA released from the spore suspensions of the CP, MF, CK, and MF + CK groups were 7.35 μmol/L, 8.83 μmol/L, 7.89 μmol/L, and 10.3 μmol/L, respectively. The results indicate that the release of DPA was significantly higher (*p* < 0.05) in the germinated MF and MF + CP groups compared to the CK and CP groups. This suggests that the germination treatment can induce the release of DPA from spores, leading to a significant increase (*p* > 0.05) in the DPA concentration of the treated spore suspensions, consistent with previous findings [32]. The DPA content in the CP and CK groups showed no significant difference (*p* > 0.05) and was lower than (*p* < 0.05) in the MF group. This indicates that cold plasma treatment alone may not be sufficient to completely release DPA from spores in the form of disrupted spore nuclei, nor induce a large number of spore germinations. Furthermore, the previous studies demonstrated that cold plasma treatment significantly reduced (*p* < 0.05) the number of viable *B. licheniformis* in the spore suspensions. This suggests that cold plasma treatment may damage spore structures beyond the core, impeding the normal processes of germination and growth, ultimately leading to spore inactivation or death. The higher release of DPA in the MF + CP group compared to the MF group may be attributed to incomplete DPA release during spore germination and a partial reduction in heat resistance, followed by the release of all remaining DPA after inactivation via cold plasma treatment [8,32]. Therefore, further studies are necessary to confirm the disruption of spore structures by cold plasma.

The inner membrane of spores plays a critical role in maintaining their high resistance and protecting the spore core from harmful environmental chemicals due to its low permeability to small molecules and water. The inner membrane also contains numerous receptors associated with germination. Disruption of this membrane can result in an osmotic pressure imbalance, which hinders normal growth and germination. Figure 3E shows significant variations in the colony morphology of spores when grown in a normal medium and two hypertonic media. The size, shape, and transparency of the colonies differed markedly among the different media, indicating that the spores responded to the changes in their environment. In the NA medium, the vegetative cells germinated from spores exhibited vigorous growth with a distinct colony morphology characterized by irregular edges and high transparency. In contrast, the vegetative cells germinated from spores grown in an NA medium containing 1 mol/L NaCl formed smaller, rounder colonies with reduced transparency and large individual colonies. The addition of 50 mmol/L glucose to the hypertonic medium resulted in the smallest colonies with a rounded shape. The results suggested that undamaged spores can still germinate into vegetative cells and then grow in hypertonic media. However, the observed changes in colony morphology indicate that the hypertonic environment has a significant impact on the growth and development of vegetative cells germinated from spores. The number of surviving spores in the CP and MF + CP treatment groups was significantly lower (*p* < 0.05) in the normal medium compared to the CK and MF groups (*p* < 0.05), and there was also a significant difference (*p* < 0.05) between the CP and MF + CP groups (Table 2). These results were consistent with previous findings. When spore suspensions were incubated in an NA medium containing 1 mol/L NaCl, the survival rate of spores significantly decreased (*p* < 0.05) in the groups treated with germination, cold plasma, or their combination, while there was no significant difference (*p* > 0.05) observed in the untreated CK group. This indicates that untreated spores can still germinate and grow in hypertonic media, whereas treatments that induce germination, cold plasma treatments, or their combinations all impact spore survival in hypertonic environments. These findings are consistent with previous studies reporting that germinated spores are less resistant to salt than non-germinated ones [33]. In contrast, the survival rate of cold-plasma-treated spores was 49.78% lower in the hypertonic medium containing 1 mol/L NaCl compared to the NA medium (Table 2), indicating that the cold plasma treatment impairs the spores’ ability to maintain osmotic pressure equilibrium. Similarly, vegetative cell growth was inhibited in a hypertonic medium supplemented with 50 mmol/L glucose, although partial recovery was observed compared to growth in an NA medium containing only 1 mol/L NaCl. However, the effect was not significantly different (*p* > 0.05). This partial recovery might be attributed to the spores’ self-healing ability, which is beyond the scope of the present study. Our findings suggested that ungerminated spores can survive in hypertonic media, while germinated spores are more susceptible to salt stress. Furthermore, cold plasma treatment appears to decrease spore survival in hypertonic environments, possibly through disruption of the spores’ inner membrane.

The inner membrane of the spores has a low permeability to propidium iodide (PI) dye. This means that PI stain can only attach to the DNA in the inner core and produce a red fluorescence when the inner membrane is disrupted [34]. To assess the integrity of a spore’s inner membrane, observation using a confocal laser scanning microscope (CLMS) was conducted. Figure 3F presents laser confocal images of spore suspensions stained with PI fluorescence after undergoing different treatments. Untreated spores do not exhibit any fluorescence, indicating that spores with intact inner membranes are not susceptible to PI staining. However, after germination and treatment at 80 °C, distinct rod-like red fluorescence was observed under confocal microscopy. This result suggested that the “A”GFNa-1 induced germination of *B. licheniformis* spores, and the resulting morphological changes gave rise to an irregular rod-like pattern under laser microscopy. In the CP group, only punctate red fluorescence was observed in the laser confocal images, indicating that cold plasma disrupts the inner membrane structure of spores, allowing PI to bind to DPA, but does not act by first germinating spores and then inactivating them. In contrast, the MF + CP group exhibited both rod-like and dot-like red fluorescence, suggesting that cold plasma cannot only destroy the inner membrane of unsprouted *B. licheniformis* spores but also inactivate their vegetative cells. These results were consistent with previous findings [23]. Although techniques like laser confocal microscopy have demonstrated that cold plasma can disrupt the spore inner membrane and partially inactivate spores, the exact mechanism by which cold plasma disrupts the inner membrane remains unclear. Further research is necessary to elucidate this precise mechanism.

## 4. Conclusions

In our study, we selected a novel bactericidal combination: the germinant “A”GFNa-1 combined with a 70 kV cold plasma. We observed a synergistic effect between the cold plasma treatment and the germinant “A”GFNa-1, resulting in an impressive inactivation rate of 99.20% for both *B. licheniformis* spores and vegetative cells. Thermal inactivation and DPA release rate results confirmed that cold plasma effectively inactivates *B. licheniformis spores* and vegetative cells, without inducing germination. Furthermore, the survival rate of spores in a hypertonic medium and the punctate fluorescence observed through GLSM indicated that the inactivating effect of cold plasma is not dependent on the germinating agent, but instead on the disruption of the inner membrane structure of the spores. These findings suggest that combining an efficient germinant with cold plasma treatment shows promise as a viable approach for controlling spore contamination in the food industry.

## Figures and Tables

**Table 1:** The chemical composition and concentration of germinants.

Germinants	Formulation Concentration	
D-glucose (G)	30 mmol/L 40 mmol/L 50 mmol/L 60 mmol/L 70 mmol/L	
D-fructose (F)		
D-galactose (Gal)		
L-Alanine (Ala)		
L-Asparagine (Asp)		
L-Valine (Val)		
L-Proline (Pro)		
L-Lysine (Lys)		
L-Histidine (His)		
Inosine	4 mmol/L 6 mmol/L 8 mmol/L	
DPA ^1^		
AGFK	Asp (10 mmol/L) G (10 mmol/L) F (10 mmol/L) KCl (50 mmol/L)	
Combined germinants “A”GFK/Na/Mg/Ca	Ala (60 mmol/L) G (10 mmol/L) F (10 mmol/L) KCl/NaCl/MgCl_2_/CaCl_2_	0.05 g/L
		0.1 g/L
		0.5 g/L
		1 g/L
		5 g/L

^1^ DPA: Dipicolinic acid.

**Table 2:** Number of spores surviving (*n* = 3) in hypertonic media.

Treatment	Number of Spores Remaining on Different Media (log CFU/mL)		
	NA ^5^	NA + 1 mol/L NaCl	NA + 1 mol/L NaCl + 50 mmol/L Glucose
CK ^1^	3.46 ± 0.03 ^Aa^	3.39 ± 0.07 ^Aa^	3.22 ± 0.07 ^Aa^
MF ^2^	3.38 ± 0.09 ^Aa^	2.44 ± 0.1 ^Bc^	2.78 ± 0.1 ^Bb^
CP ^3^	2.23 ± 0.13 ^Ba^	1.12 ± 0.1 ^Cb^	1.22 ± 0.12 ^Cb^
MF + CP ^4^	1.39 ± 0.1 ^Ca^	0.98 ± 0.06 ^Dc^	1.27 ± 0.13 ^Cb^

^1^ CK: No germination, no cold plasma. ^2^ MF: Germination only, no cold plasma. ^3^ CP: No germination, 100% argon packed, 70 kV cold plasma for 3 min. ^4^ MF + CP: Germination for 5 h, 100% argon packed, 70 kV cold plasma for 3 min. ^5^ NA: Nutritional agar. ^a,b,c^ Different lowercase letters indicate significant differences in different culture conditions under the same treatment (*p* < 0.05). ^A,B,C^ Different capital letters indicate significant differences in different treatments under the same culture conditions (*p* < 0.05).

## Data Availability

Data are contained within the article.
